# Applied statistical training to strengthen analysis and health research capacity in Rwanda

**DOI:** 10.1186/s12961-016-0144-x

**Published:** 2016-09-29

**Authors:** Dana R. Thomson, Muhammed Semakula, Lisa R. Hirschhorn, Megan Murray, Vedaste Ndahindwa, Anatole Manzi, Assumpta Mukabutera, Corine Karema, Jeanine Condo, Bethany Hedt-Gauthier

**Affiliations:** 1 College of Medicine and Health Sciences, University of Rwanda, Kigali, Rwanda; 2 Department of Global Health and Social Medicine, Harvard Medical School, Boston, MA United States of America; 3 Ministry of Health, Rwanda Biomedical Center, Kigali, Rwanda; 4 Ariadne Labs, Boston, MA United States of America; 5 Partners in Health-Rwanda, Rwinkwavu, Rwanda; 6 Quality and Equity Healthcare, Kigali, Rwanda; 7 Swiss Tropical and Public Health Institute, Basel, Switzerland

**Keywords:** Deliverable-driven, Capacity building, Capacity strengthening, Research, Evaluation, Analysis, Statistics

## Abstract

**Background:**

To guide efficient investment of limited health resources in sub-Saharan Africa, local researchers need to be involved in, and guide, health system and policy research. While extensive survey and census data are available to health researchers and program officers in resource-limited countries, local involvement and leadership in research is limited due to inadequate experience, lack of dedicated research time and weak interagency connections, among other challenges. Many research-strengthening initiatives host prolonged fellowships out-of-country, yet their approaches have not been evaluated for effectiveness in involvement and development of local leadership in research.

**Methods:**

We developed, implemented and evaluated a multi-month, deliverable-driven, survey analysis training based in Rwanda to strengthen skills of five local research leaders, 15 statisticians, and a PhD candidate. Research leaders applied with a specific research question relevant to country challenges and committed to leading an analysis to publication. Statisticians with prerequisite statistical training and experience with a statistical software applied to participate in class-based trainings and complete an assigned analysis. Both statisticians and research leaders were provided ongoing in-country mentoring for analysis and manuscript writing.

**Results:**

Participants reported a high level of skill, knowledge and collaborator development from class-based trainings and out-of-class mentorship that were sustained 1 year later. Five of six manuscripts were authored by multi-institution teams and submitted to international peer-reviewed scientific journals, and three-quarters of the participants mentored others in survey data analysis or conducted an additional survey analysis in the year following the training.

**Conclusions:**

Our model was effective in utilizing existing survey data and strengthening skills among full-time working professionals without disrupting ongoing work commitments and using few resources. Critical to our success were a transparent, robust application process and time limited training supplemented by ongoing, in-country mentoring toward manuscript deliverables that were led by Rwanda’s health research leaders.

## Background

Healthcare needs and program impacts in sub-Saharan Africa must be appropriately studied to support efficient investment of limited health resources [1–3]. Leadership of individuals from local and national programs, for example, ministries of health, schools of public health, and non-governmental organizations, is essential to identify health research priorities, ensure that the study design and interpretation of results are locally relevant, and support the use of data in policy and programmatic decisions [4–8]. However, there are several challenges to local leadership and involvement in research, including a limited number of individuals dedicated to health research [4], limited time to incorporate research into already demanding work schedules [9], limited financial support for research [8, 10], weak interagency connections [4], weak links between curricula and competencies needed for research [11], and limited experience completing or leading health research [6, 12].

Limited research capacity in sub-Saharan African countries spans a variety of competencies, including statistical capacity to analyse data [4, 13, 14]. While countries in sub-Saharan Africa have undergraduate training in statistics and extensive raw data are available for analysis [15], there are limited options for mentorship or advanced statistics training. Initiatives to address this gap include university consortiums that place international visiting faculty in African universities and prolonged trainings outside of Africa [16]. There are few details available publicly on these interventions and limited evaluation of their effectiveness [7].

Junior researchers in sub-Saharan Africa have limited opportunities for research mentorship. Promising mentorship approaches support learners to stay in their jobs [9, 13] while benefiting from hands-on training [17] and peer-to-peer learning [18]. These approaches are supported by adult learning theory, which recommends instructor orientation followed by self-directed project-based learning [19]. To address needs for statistical capacity in Rwanda, a network of researchers linked through other research collaborations [20] developed and implemented a deliverable-driven, survey analysis training based in Rwanda in 2013. The training utilized existing data, and aimed to strengthen research skills and leadership within and across our institutions. In this paper, we describe the development and implementation of this training program and the outcomes from the first implementation.

## Methods

In 2013, two applied demography and biostatistics researchers from Harvard Medical School (DRT, BHG) and the University of Rwanda – College of Medicine and Health Sciences – School of Public Health (UR-CMHS-SPH) (BHG) coordinated a deliverable-driven training for Rwanda-based statisticians and research leaders. The course culminated in two deliverables which occurred in two phases. The first deliverable was to analyse and present statistical results following 6 days of in-class lectures and exercises and 3 weeks of one-on-one meetings. The second deliverable was to write a paper with statistical and writing mentorship from the instructors and submit it to a peer-reviewed journal within 4 months. We evaluated the quality and impact of the 6-week training on learner skills and knowledge and professional network-building, as well as the overall effectiveness for timely submission of quality manuscripts.

### Participant selection

An announcement, application forms [21], and the 2010 Rwanda Demographic and Health Survey (RDHS) final report were emailed 1 month before the application deadline to researchers, program leaders, statisticians and graduate students at the Ministry of Health in Rwanda, UR-CMHS-SPH, Rwanda Biomedical Center, National Institute of Statistics –Rwanda, and Partners in Health – Rwanda (PIH). Applicants from other institutions were also welcomed to apply.

Two types of applicants were solicited. Project leader applicants provided a brief research proposal and their curriculum vitae. Research proposals were evaluated for feasibility and relevance, and project leaders were assessed for ability to formulate a clear question and organize ideas in writing. Instructors worked with these applicants to refine research questions that could be answered using logistic regression modelling (the focus of the training). Project leaders committed to leading a research team during the training and completing a scientific manuscript in the 4 months following the training.

Statistician applicants were required to have taken at least two introductory biostatistics courses and have used a statistical software package. In their application, they described the relevance of the training to their work or research and provided a letter of recommendation from a supervisor. Statisticians committed to attending all in-class training sessions and to working with a team to analyse an assigned research question.

All applications were pre-screened and scored [21] by both instructors. A selection committee comprised of leaders from invited institutions (MS, LRH, MM, JC) reviewed the applications and made the final selection. Preference was given to applicants who could apply the training directly to their work, with efforts to balance representation of organisations and genders.

### Curriculum

Lectures and exercises guided teams to download datasets, define variables, perform bivariate and multivariate statistics, and interpret results. Since most participants had to take leave from full-time jobs to attend trainings, the curriculum was condensed into six all-day sessions with 3 consecutive days in weeks 1 and 3. To reduce distractions and costs, sessions were held at a rurally located training centre operated by PIH.

Materials included a binder with lecture notes, practice exercises, Stata software, EdX videos reviewing Stata commands, datasets, example statistical code, example methods write-up, articles about Demographic and Health Surveys (DHSs), and a USB with all aforementioned materials. Participants were offered transport, sleeping accommodation, materials and meals, but no per diems. Each participant had to complete daily assignments during sessions, complete a statistical analysis, and deliver a presentation to be eligible for a certificate.

### Costs

Instructor time, including for curriculum development, was provided by Harvard Medical School Department of Global Health and Social Medicine – Global Health Research Core. The training venue in Rwanda was provided in-kind by PIH. All other expenses, including course materials, Stata licenses, textbooks, instructor travel, and participant meals and transport, totalling US$ 9300 were covered by the Doris Duke Charitable Foundation Africa Health Initiative.

### Evaluation

For each of the 12 lectures, learners were asked to rate improvement in knowledge and likelihood to use the information, tools or skills in their work. Responses ranged from 1 (not at all) to 4 (very much). On the last training day, learners completed an overall evaluation of 13 areas of professional growth and relationship building, and were asked to rate the same 13 areas in a 1-year follow-up survey. The end-of-course evaluation and the 1-year follow-up had several open-ended questions about favourite parts of the training, parts to change and recommendations to improve effectiveness. In the 1 year follow-up, participants were asked about their engagement with manuscript writing, and other DHS or survey analyses [21].

### Ethics review

All research questions used secondary data from RDHS, which has existing ethics clearance from the Rwanda National Ethics Committee. This evaluation of the training was reviewed and approved by the UR-CMHS Internal Review Board (Ref: UR-CMHS/IRB/006/2015).

## Results

### Participants

Of the 10 team leader applicants, five were selected with distinct, answerable research questions, and a PhD student was selected as a sixth project leader to work independently on a dissertation paper. While team leaders were not required to attend the training, all opted to fully participate. Of the 40 statistician applicants, 16 were selected and assigned to one of five teams. Seven women and 14 men were selected from the Ministry of Health (1), National University of Rwanda School of Public Health (5), National Institute of Statistics Rwanda (4), PIH (2), Rwanda Biomedical Center (7), the Centers for Disease Control and Prevention – Rwanda (2), and one person was a consultant (1).

### Course evaluation

All except one statistician successfully completed the 6-week course. Of the 21 participants who completed the course, 16–20 provided anonymous daily feedback on lectures, 16 responded to the final course evaluation, and 14 completed the 1-year follow-up survey. All lectures received a median score of 3 or 4 indicating that most learners felt the lectures improved their knowledge “quite a bit” or “very much” (Table 1). Most learners said they were “very much” likely to apply the lecture content in their work, with the exception of conceptual frameworks.

**Table 1:** Scores for individual lectures

Lecture	n	Lecture improved knowledge^a^	Likelihood of using information/tool/skill in work^a^
		Median (range)	Median (range)
1. Simple random sampling	20	4 (2–4)	4 (2–4)
2. Complex sampling	20	4 (2–4)	4 (2–4)
3. Demographic health survey (DHS) sample design; DHS documents	17	4 (2–4)	4 (3–4)
4. DHS in Stata	17	3 (2–4)	4 (3–4)
5. Generating variables; Summary stats	17	3 (2–4)	4 (2–4)
6. Conceptual framework; Constructs	17	3 (2–4)	3 (3–4)
7. Table 1	16	4 (3–4)	4 (3–4)
8. Univariable regression	16	4 (3–4)	4 (3–4)
9. Correlation; Collinearity	16	4 (3–4)	4 (3–4)
10. Correlation; Collinearity application	17	4 (2–4)	4 (3–4)
11. Multivariable regression	17	3 (1–4)	4 (3–4)
12. Multivariable regression application	17	3 (2–4)	4 (3–4)

^a^Scores: 1 – not at all, 2 – a little, 3 – quite a bit, 4 – very much

The training had positive impacts on multiple areas of skills, knowledge and relationship building. At the end of the course and after 1 year, the median respondent said the training had “very much” improved their ability to understand statistical results and to complete analysis for publication (Table 2). Other impacts included generation of ideas for future research, abilities to use Stata for analysis, explain analysis to others, complete logistic regression, and communicate about data and research (median score 3.5 for all after 1 year). In the 1 year following the training, 36% of participants completed an additional DHS analysis, 71% completed an additional survey analysis, and 79% provided mentorship to others about survey data analysis.

**Table 2:** Overall course evaluations, median score (range)

Feedback (ordered from highest-to-lowest score)	End of course^a^	1-year follow-up^a^
*How much did this DHS training improve your …*		
Understanding of results from statistical analysis	4 (2–4)	4 (2–4)
Ability to complete analysis which could contribute to a publication	4 (1–4)	4 (2–4)
Generation of ideas of future research	4 (3–4)	3.5 (2–4)
Ability to use Stata for analysis	4 (2–4)	3.5 (2–4)
Ability to explain analysis to others	4 (2–4)	3.5 (2–4)
Ability to complete logistic regression	4 (2–4)	3.5 (1–4)
Ability to communicate about data and research	4 (1–4)	3.5 (2–4)
Ability to complete data analysis in complex survey data	4 (3–4)	3 (2–4)
Network with other colleagues in Rwanda	4 (3–4)	3 (2–4)
Establishment of future research collaborations	4 (3–4)	3 (2–4)
Identification of resources for future research	3.5 (2–4)	3 (1–4)
Use of data to support program management/policy	3.5 (1–4)	3 (1–4)
Ability to build a statistical model	3 (2–4)	3 (1–4)
*How useful were the following materials/activities?*		
Hand-out of slides with text (no room for notes)	4 (3–4)	3 (2–4)
Demographic and Health Survey (DHS) pre-downloaded datasets	4 (1–4)	3 (2–4)
Hand-out of slides with room for notes (no additional text)	4 (3–4)	3 (1–4)
Example .do files	4 (2–4)	3 (1–4)
Stata	4 (2–4)	3 (1–4)
Practice exercises (standardized across all groups)	4 (2–4)	3 (1–4)
Binders of materials	4 (1–4)	3 (1–4)
Materials on USB drives	4 (1–4)	3 (1–4)
EdX videos on Stata	3 (2–4)	3 (1–3)
Practice exercises (group specific)	4 (2–4)	2.5 (1–4)
Pre-training readings on DHS	4 (2–4)	2.5 (1–4)
Pre-training readings on topics	4 (2–4)	2.5 (1–4)
Example DHS methods write-up	4 (2–4)	2.5 (1–4)
N	16	14

^a^Scores: 1 – not at all, 2 – a little, 3 – quite a bit, 4 – very much

Overall, learners found all provided tools and materials were very useful during the course except for the EdX videos (Table 2). One year after the course, the lecture hand-outs with text and the pre-downloaded datasets were considered “quite a bit” useful by most learners, and lecture hand-outs with personal notes, example statistical code, Stata software, generic practice exercises and pre-packaged binder or USB with materials were “quite a bit” useful to some learners.

At the end of the course, most learners described the training as “incredible” or “fantastic” and these adjectives persisted a year later in open-ended comments. However, numerous comments also described the training’s “intensity”. Recommendations included organising writing workshops during the paper-writing phase, better linking development of research questions to policy priorities, increasing the number of days for training and data analysis, and organising additional trainings using census, national health management information systems, and routinely-collected hospital data.

### Paper deliverables

No teams submitted a manuscript within 4 months; the first manuscript was submitted within 6 months, two additional manuscripts were submitted within 12 months, a fourth manuscript was submitted at 18 months, and a fifth manuscript was submitted at 26 months to international peer-reviewed journals. At the time of this writing, three manuscripts were published [22–24], two were under revision to resubmit, and one remained under development.

### Other networking and analysis activities

Of the 14 participants who responded to the 1-year follow-up survey, most reported having been actively engaged in the manuscript writing process by participating in face-to-face or phone meetings with the course facilitators (*n* = 9, 64%) or other participants (*n* = 11, 79%) (Table 3). In the 1 year after the course, most participants also mentored others through DHS or survey data analysis (*n* = 11, 79%), and many also performed their own additional DHS (*n* = 5, 36%) or other survey (*n* = 10, 71%) analyses.

**Table 3:** Networking and research activities after the training

Have you… ______ … after the training?	Count	(%)
Worked directly (face-to-face/phone) with course facilitators on your DHS paper	9	(64)
Worked directly (face-to-face/phone) with other course participants on your DHS paper	11	(79)
Been involved with additional DHS analyses	5	(36)
Been involved with additional survey data analyses	10	(71)
Provided mentorship to others about DHS or survey data analysis	11	(79)
Total	14	

## Discussion

This evaluation of a deliverable-driven statistical analysis training in Rwanda found positive, lasting impacts on data analysis skills and knowledge, as well as sustained inter-institutional relationships. We summarize six lessons learned about the training approach which may be applicable to health research capacity-strengthening in other resource-limited settings. We provide our application, selection and evaluation materials in case readers wish to adapt them [21].

1. Set concrete milestones with realistic timelines
  This deliverable-driven training successfully advanced statistical skills, knowledge and relationship building among Rwandan researchers through an in-country experience with minimal days away from work. This was possible over 2 months with concrete deliverables and face-to-face support throughout the following year, making this deliverable-driven training a viable alternative to prolonged out-of-country trainings. Our timeline for deliverable 2 was overly optimistic; a 1-year timeline with regularly scheduled writing retreats and interim deliverables would have been more realistic.
2. Integrate training into work schedules
  For everyone in the training, research was only a part of their work responsibilities, if at all, so the time-intensive task of manuscript writing required extensive effort outside of work for months following the training. Participants recommend that, in the future, we should organize writing workshops to ensure scientific writing support and mentorship, as well as to protect time for scientific writing.
3. Provide ongoing mentorship
  All papers required more on-going, in-country support from the instructors than initially expected because all papers had a first-time first author for whom English was not their first language. The instructors led follow-up with three teams each, with in-person meetings every 1–3 weeks as needed. The instructors met monthly to share updates of their teams’ progress and were able to stand-in for one another during extended periods of leave. In the 2 years since the end of the training, the instructors had more than 150 hours of face-to-face meetings with first and/or senior authors to provide nuanced, supportive, critical feedback, and to keep the manuscript a priority among many other competing priorities. Formal and informal face-to-face access to research mentors throughout the analysis and writing phases were critical for professional development, and this would have been extremely difficult if instructors were not based in-country. The high value of face-to-face personalized mentorship was likely reflected in lower scores for EdX Stata videos compared to other training materials.
4. Rigorous selection
  A rigorous application process ensured high-calibre learners who invested themselves in the training, and did not view the training as a requirement or a break from work. The high completion rate, and proportion of training participants involved with survey data analysis and mentorship in the 1-year following the training suggests that a deliverable-driven training model can promote involvement of Africans in African-based research [5] and foster research leadership.
5. Invest in proper materials
  Access to software and high-quality reference materials are key to the success of any researcher, especially researchers with limited bandwidth and research support outside of training. If the training fails to provide a statistical software license, lecture notes and other reference material, it is unlikely that the researcher will be able to obtain these resources on their own.
6. Harmonize with local institutions
  Having an instructor based in Rwanda familiar with in-country institutional priorities was key toward refining and selecting research questions during the application process that could strengthen local institutional capacity. In the absence of that, syllabus development and topic and trainee selection must be advised heavily by stakeholders in the field. We strongly recommend that deliverable-driven initiatives be designed and taught by national instructors, and nationally-based foreign instructors (if needed), rather than by foreign-based instructors. This might mean that national universities second the time of their faculty to build local capacity via such trainings.

### Limitations

Limitations of this evaluation should be considered when interpreting results. First, this was a small sample from one course conducted in the context of an existing partnership among several academic, policy and health intervention institutions. Additional considerations may be needed in other partnerships such as availability of resident instructors and mentors. Second, results were self-reported. Finally, five (24%) and seven (33%) participants did not respond to the end-of-course and 1-year follow-up surveys, respectively. Results may be biased toward affirmative responses.

## Conclusions

This deliverable-driven training was part of a larger research capacity strengthening initiative in Rwanda involving the aforementioned institutions [3], and informed other activities similarly designed to reinforce didactic material with application to research projects while receiving mentorship. These subsequent activities include a deliverable-driven seminar series for junior faculty at UR-CMHS-SPH to publish their first international research manuscript (resulting in five published papers), a multi-month training across multiple research institutions using time-series analysis (resulting in two papers still under review), a practice-based survey analysis course at UR-CMHS-SPH based on this curriculum (taught three times, most recently by a participant of this training), and three intermediate operational research courses targeting district-based PIH and Ministry of Health trainees (resulting in seven published papers, with another 13 under development). Deliverable-driven trainings paired with learning material and in-country mentorship can be a source of critical statistical skill and leadership development for participating individuals and their institutions.

## Abbreviations

PIH: Partners in Health – Rwanda
RDHS: Rwanda Demographic and Health Survey
UR-CMHS-SPH: University of Rwanda – College of Medicine and Health Sciences – School of Public Health
